# Hypoxic Characteristic in the Immunosuppressive Microenvironment of Hepatocellular Carcinoma

**DOI:** 10.3389/fimmu.2021.611058

**Published:** 2021-02-17

**Authors:** Zhuomao Mo, Daiyuan Liu, Dade Rong, Shijun Zhang

**Affiliations:** ^1^Department of Traditional Chinese Medicine, The First Affiliated Hospital, Sun Yat-sen University, Guangzhou, China; ^2^Department of Biochemistry, Zhongshan School of Medicine, Sun Yat-sen University, Guangzhou, China

**Keywords:** hepatocellular carcinoma, hypoxia, score, immunotherapy, tumor microenvironment

## Abstract

**Background:** Generally, hepatocellular carcinoma (HCC) exists in an immunosuppressive microenvironment that promotes tumor evasion. Hypoxia can impact intercellular crosstalk in the tumor microenvironment. This study aimed to explore and elucidate the underlying relationship between hypoxia and immunotherapy in patients with HCC.

**Methods:** HCC genomic and clinicopathological datasets were obtained from The Cancer Genome Atlas (TCGA-LIHC), Gene Expression Omnibus databases (GSE14520) and International Cancer Genome Consortium (ICGC-LIRI). The TCGA-LIHC cases were divided into clusters based on single sample gene set enrichment analysis and hierarchical clustering. After identifying patients with immunosuppressive microenvironment with different hypoxic conditions, correlations between immunological characteristics and hypoxia clusters were investigated. Subsequently, a hypoxia-associated score was established by differential expression, univariable Cox regression, and lasso regression analyses. The score was verified by survival and receiver operating characteristic curve analyses. The GSE14520 cohort was used to validate the findings of immune cell infiltration and immune checkpoints expression, while the ICGC-LIRI cohort was employed to verify the hypoxia-associated score.

**Results:** We identified hypoxic patients with immunosuppressive HCC. This cluster exhibited higher immune cell infiltration and immune checkpoint expression in the TCGA cohort, while similar significant differences were observed in the GEO cohort. The hypoxia-associated score was composed of five genes (ephrin A3, dihydropyrimidinase like 4, solute carrier family 2 member 5, stanniocalcin 2, and lysyl oxidase). In both two cohorts, survival analysis revealed significant differences between the high-risk and low-risk groups. In addition, compared to other clinical parameters, the established score had the highest predictive performance at both 3 and 5 years in two cohorts.

**Conclusion:** This study provides further evidence of the link between hypoxic signals in patients and immunosuppression in HCC. Defining hypoxia-associated HCC subtypes may help reveal potential regulatory mechanisms between hypoxia and the immunosuppressive microenvironment, and our hypoxia-associated score could exhibit potential implications for future predictive models.

## Introduction

As the major subtype of liver cancer, hepatocellular carcinoma (HCC) is diagnosed in more than half a million people worldwide each year (1). Characterized by high metastasis and poor prognosis, HCC is one of the most common causes of cancer death (2). Curative treatments, including liver transplantation, liver resection, and ablation are preferred for HCC (3); however, most patients are not suitable for curative treatment, since they are usually diagnosed at advanced stages. In addition, although systemic therapy with sorafenib is the first-line chemotherapeutic treatment for patients with advanced HCC, most patients are highly refractory to this therapy (4). Therefore, there is an urgent need to investigate novel treatments to improve the prognosis of most patients with HCC.

Malignant tumor tissues include not only tumor cells, but also supportive tumor-associated healthy cells (stromal cells and immune cells), which comprise the tumor microenvironment (TME) (5). The TME has recently attracted increasing attention, as it provides a novel context for tumor diagnosis and prognosis (6). The TME also provides essential cues to maintain stemness and promote the seeding of tumor cells at sites of metastasis (5). The estimation of stromal and immune cells in malignant tumors using expression data (ESTIMATE) algorithm can be used to estimate and quantify the TME. Many studies (6–8) have shown that stromal score, immune score, and tumor purity measurements based on the TME can serve as prognostic tumor biomarkers. For HCC, immunohistochemical scoring of CD38 molecule in the TME can be used to predict responsiveness to anti-programmed cell death 1/CD274 molecule (i.e., anti-PD-1/PD-L1) immunotherapy (9). In addition, a TME-based risk score was shown to be an effective prognostic predictor for HCC (10). However, the HCC TME is complex, with diverse populations of innate and adaptive immune cells that influence tumor immune evasion and the response to immunotherapy (11). Furthermore, the HCC TME is characterized by the presence of multiple immunosuppressive factors (12). Therefore, it is necessary to explore and elucidate the roles of intrinsic cellular factors and extrinsic factors in patients with immunosuppressive HCC TME.

Hypoxia is a typical characteristic of the TME, and drives the aggressiveness of many tumors (13–15). In the process of adapting to the hypoxic TME, cancer cells acquire invasive and metastatic properties (16). Interestingly, this hypoxia-associated signature has impressive pan-cancer predictive potential (17). Hypoxia regulates the mitochondrial activity of HCC cells through the hypoxia-inducible factor (HIF)/hes related family bHLH transcription factor with YRPW motif 1/PTEN induced kinase 1 pathway (13). Another study found that the hypoxia-induced microRNA miR-3677-3p promoted the proliferation, migration, and invasion of HCC cells by suppressing sirtuin 5 (18). Moreover, hypoxia inducible lipid droplet associated promotes HCC immune escape from natural killer cells through the interleukin 10/signal transducer and activator of transcription 3 signaling pathway (19). These studies demonstrate that hypoxia plays a crucial role in HCC immunotherapy. However, the underlying mechanism remain to be investigated.

In this study, we first used 29 immune-associated gene sets to identify patients with immunosuppressive TME of HCC through hierarchical clustering. Next, using the same algorithm and clustering method, we identified a hypoxic cluster among the immunosuppressive patients. Patients in the hypoxia group had higher immune cell infiltration and immune checkpoint expression, suggesting increased sensitivity to immunotherapy. Furthermore, we established a hypoxia-related score to predict the prognosis of patients with immunosuppressive HCC. Our results indicate that the presence of TME hypoxia is a potential biomarker of HCC immunotherapeutic response and prognosis.

## Materials and Methods

### Data Collection

Gene expression and clinical data were retrieved from The Cancer Genome Atlas (TCGA; https://portal.gdc.cancer.gov/), Gene Expression Omnibus (GEO; https://www.ncbi.nlm.nih.gov/geo/) and International Cancer Genome Consortium (ICGC; https://dcc.icgc.org/) databases. Three independent cohorts (TCGA-LIHC, GSE14520, and ICGC-LIRI) were employed in our research, with the TCGA-LIHC cohort used as a training dataset and the other two cohorts used as a validation dataset. The hypoxia-associated gene set (Hallmark-hypoxia) was obtained from the MSigDB database (https://www.gsea-msigdb.org/gsea/index.jsp). Hypoxia-associated genes were defined as genes experimentally shown to be upregulated in response to low oxygen levels.

### Sparse Hierarchical Clustering and Cluster Validation

First, single sample gene set enrichment analysis (ssGSEA), a special type of GSEA that can estimate a score for each case, was performed using the “GSVA” package, to calculate enrichment scores based on 29 immune-related gene sets. Genes in the immune-related gene sets are shown in Supplementary File 1. Second, using the “sparcl” package in RStudio, sparse hierarchical clustering analysis was performed to identify TME with different immunological features based on the ssGSEA results. The “sparcl” package uses a novel framework for sparse clustering, in which observations are clustered based on an adaptively chosen subset of features (20). After cluster identification, the expression levels of major histocompatibility complexes, T cell inhibitors, and T cell stimulators were used to verify the distinct immunological landscapes of the identified clusters. Meanwhile, to verify the TME of different clusters, we estimated the stromal and immune scores of each case using the “ESTIMATE” package. ESTIMATE is a tool for predicting tumor purity, as it detects the presence of infiltrating stromal/immune cells in tumor tissues (21). The ESTIMATE algorithm is based on ssGSEA and generates three final scores: the stromal score (which represents the presence of stromal cells in tumor tissues), the immune score (which represents the infiltration of immune cells in tumor tissues), and the ESTIMATE score (which infers tumor purity). To further explore the hypoxic TME of patients in the immunosuppressive cluster, the expression levels of 200 hypoxia-related marker genes were used to identify different hypoxic clusters by hierarchical clustering. Clusters with different TME immunological characteristics and with different hypoxic characteristics were visualized using tree diagrams. Next, to further investigate correlations between hypoxia and immunotherapy, we examined differences in immune cell infiltration and immune checkpoint gene expression between the clusters. Immune cell infiltration was estimated using the “TIMER2.0” and “MCP-counter” methods. TIMER2.0 (http://timer.comp-genomics.org/) is a comprehensive resource for the systematic analysis of immune cell infiltration, which analyzes six types of immune cells (B cells, CD4^+^ T cells, CD8^+^ T cells, neutrophils, macrophages, and dendritic cells). MCP-counter predicts the abundance of 10 cell populations (eight types of immune cells, endothelial cells, and fibroblasts) based on the transcriptomic profiles of human tissues. Immune checkpoint genes (22, encoding both ligands and receptors) were obtained from previous studies (22).

### Generation of Hypoxia-Related Score in the Immunosuppressive Cluster

To further elucidate the underlying association between TME hypoxia and clinical HCC immunotherapy, we established a score based on hypoxia-related marker genes. First, differential expression analysis was performed to select the marker genes. Genes with both *P* < 0.05 and |log_2_fold change| > 2 were considered significantly differentially expressed. A volcano plot was used to visualize the differentially expressed genes. Subsequently, we performed univariate Cox regression analysis to further explore the prognostic genes. Genes in the univariate analysis were eligible for further selection if *P* < 0.01. Lasso regression analysis was performed to establish the hypoxia-related score. In this analysis, a lasso penalty was used to account for shrinkage and variable selection. The optimal value of the lambda penalty parameter was defined by performing 10 cross-validations. The formula for calculating hypoxia-related score was as follows: *score* = (*coefficient of mRNA*1 × *expression of mRNA*1) + (*coefficient of mRNA*2 × *expression of mRNA*2)+ ⋯ + (*coefficient of mRNAn* × *expression mRNAn*). Furthermore, to investigate the correlation between the hypoxia-related score and overall survival, we performed survival analysis using the “survival” package. The patients were divided into two groups (high-risk or low-risk group) based on the median of risk score. Correlations between the established score and other clinical parameters (age, gender, stage, tumor grade, tumor size, distant metastasis, lymph node metastasis, alpha fetoprotein, albumin, and prothrombin time) was also investigated. To further verify the hypoxia-related score, a receiver operating characteristic (ROC) curve was constructed to examine the prognostic accuracy. Meanwhile, univariate and multivariate Cox regression analyses were performed to verify whether the hypoxia-related score was an independent prognostic marker of HCC.

### Validation of Hypoxia-Related Classification and Scoring

To ensure the reliability of the established classification and score and validate the immunologic landscapes of the clusters, we repeated the clustering using the GSE14520 cohort. In addition, immune cell infiltration and immune checkpoint expression between hypoxia-related clusters were estimated and compared. Furthermore, the established score was validated by ICGC-LIRI cohort. After identifying the immunosuppressive patients, survival analysis and ROC curve were performed again.

## Results

### Identification and Validation of an Immunosuppressive HCC Cluster

**Figure 1:**
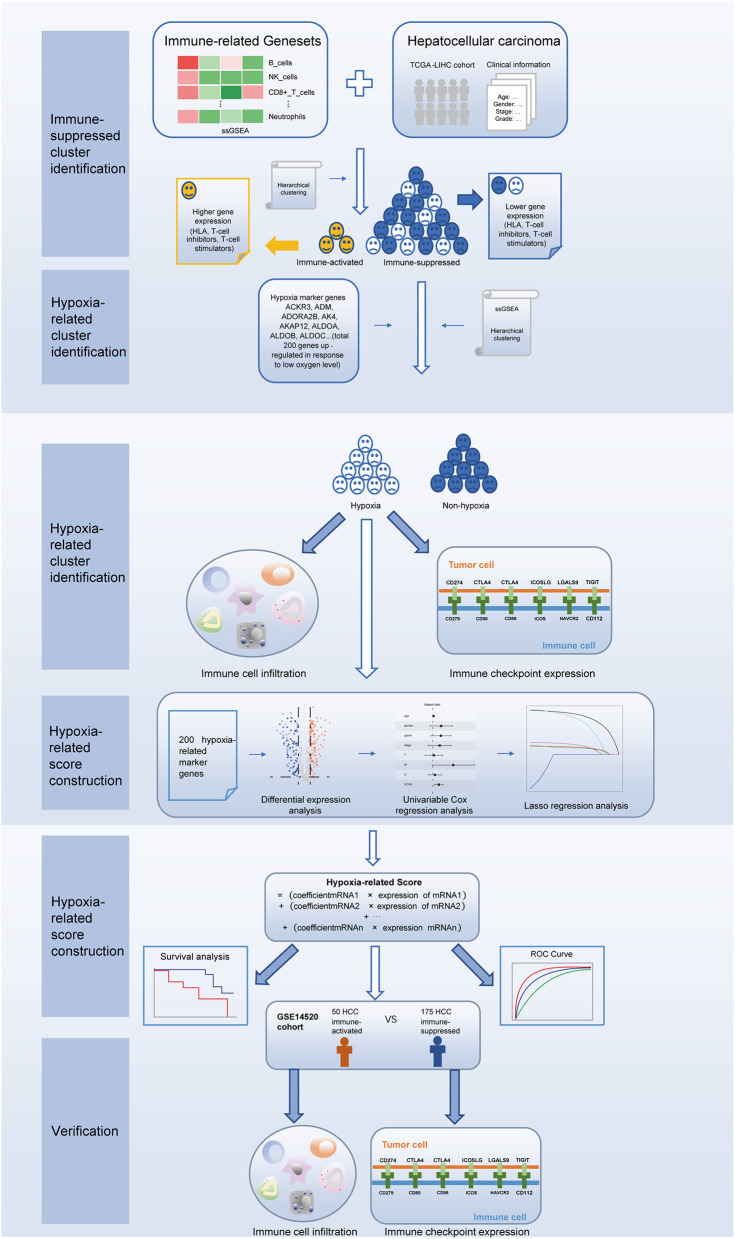
Study flowchart.

**Figure 2:**
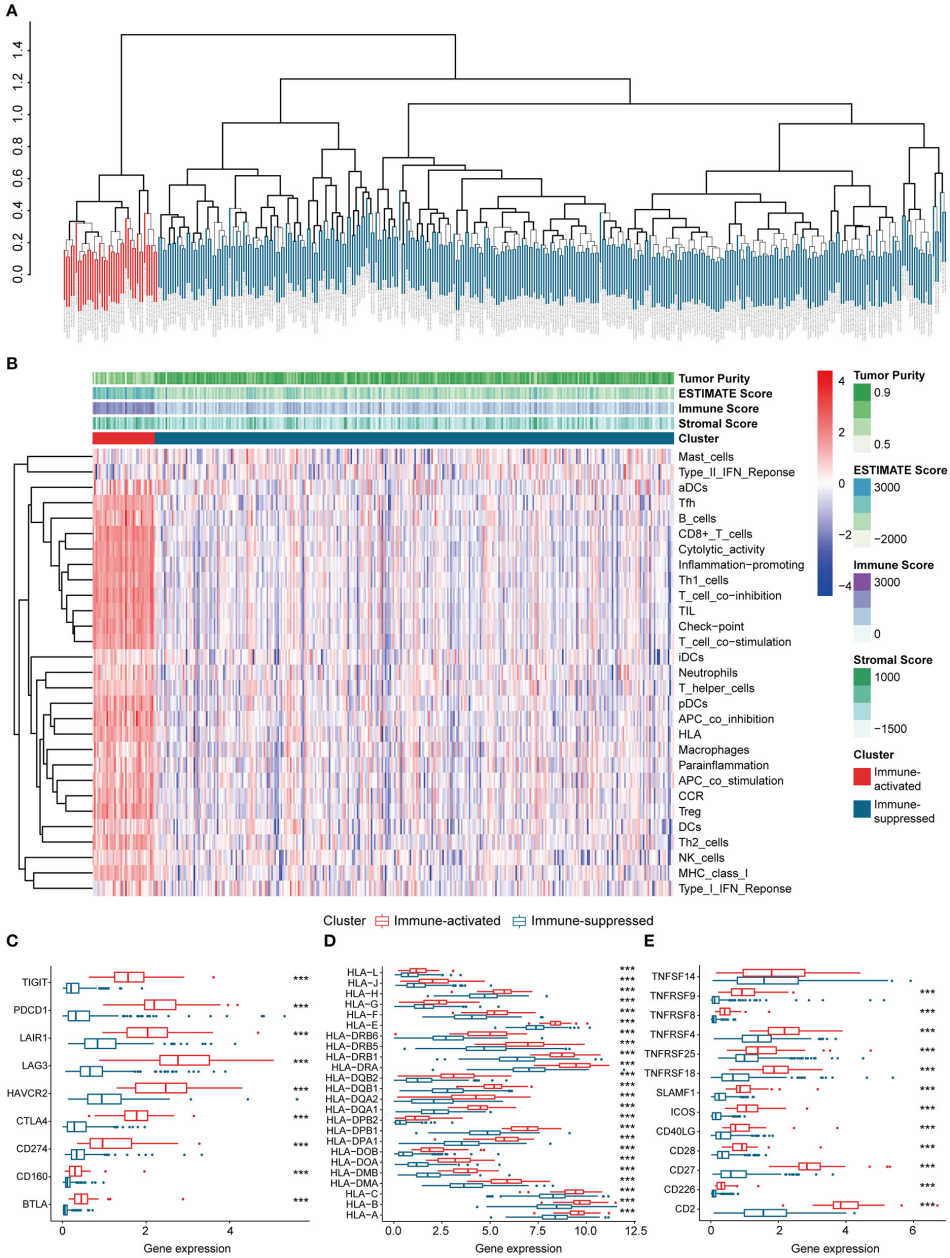
Identification and validation of immune-associated clusters **(A)** Hierarchical clustering of the immune-activated (red) and immune-suppressed (blue) clusters. Each branch in the tree diagram represents one case in the LIHC cohort. **(B)** Heatmap of immune-associated clusters, ssGSEA results, and ESTIMATE score. **(C–E)** Expression of T cell inhibitors **(C)**, major histocompatibility complexes **(D)**, and T cell stimulators **(E)** in each cluster.

The procedures in this study are summarized in Figure 1. Clinical information for the LIHC and LIRI cohorts is presented in Table 1. No relevant clinical information of GSE14520 cohort was found in GEO database. Two clusters were generated by ssGSEA and hierarchical clustering (Figure 2A). Each branch in tree diagram represented the case of LIHC cohort. The red one represented the immune-activated cluster while the blue one represented the immunosuppressive cluster. There were 40 cases in the immune-activated cluster, while the remainder comprised the immune-suppressed cluster. Figure 2B shows the ESTIMATE and ssGSEA scores of the 29 immune-related gene sets in the two clusters. Compared with the immune-activated cluster, the patients in the immunosuppressive cluster presented relatively lower immune score, lower stromal score, higher tumor purity, and lower levels of immune-related gene sets. Patients in the immunosuppressive cluster also exhibited significantly lower expression levels of T cell inhibitors (Figure 2C), major histocompatibility complexes (Figure 2D), and T cell stimulators (except for TNF superfamily member 14; Figure 2E).

**Table 1:** Baseline characteristics of patients in two cohorts.

**Clinical characteristics**		**Number**	**Percent (%)**
**TCGA-LIHC (*****n*** **=** **377)**			
Survival status	Survival	249	66
	Death	128	34
Age (1 patient missing)	≤ 65 years	235	62.5
	>65 years	141	37.5
Gender	Female	122	68
	Male	255	32
TNM Stage (24 patients missing)	I	175	50
	II	87	24.6
	III	86	24.4
	IV	5	1
Grade (5 patients missing)	G1	55	14
	G2	180	48
	G3	124	33
	G4	13	5
T classification (3 patients missing)	T1	185	49
	T2	95	26
	T3	81	22
	T4	13	3
**ICGC-LIRI (*****n*** **=** **260)**			
Survival status	Survival	214	82.4
	Death	46	17.6
Age	≤ 65 years	98	37.7
	>65 years	162	62.3
Stage	I	40	15.4
	II	117	45
	III	80	30.8
	IV	23	8.8

### Identification and Verification of a Hypoxia-Related Immunosuppressive HCC Cluster

**Figure 3:**
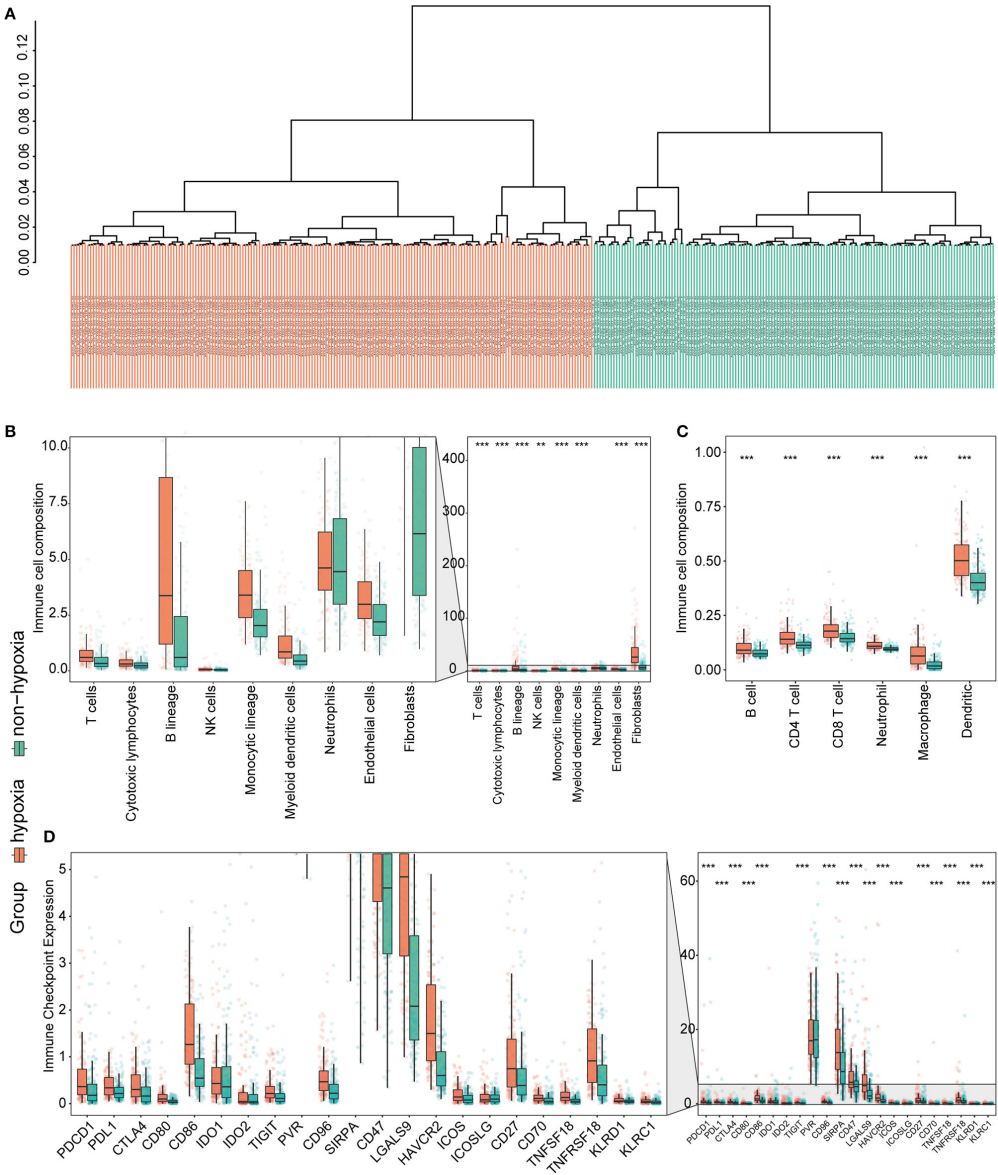
Identification and validation of hypoxia-associated clusters in the immunosuppressive cluster. *P < 0.05, **P < 0.01, ***P < 0.001. **(A)** Hierarchical clustering of hypoxic (orange) and non-hypoxic (green) patients. **(B,C)** Immune cell infiltration in the hypoxia-associated clusters based on the MCP-counter **(B)** and TIMER2.0 **(C)** methods. **(D)** Immune checkpoint gene expression in each cluster.

Considering the crucial role of hypoxia in the TME, we characterized the hypoxia observed in cases in the immunosuppressive cluster. Using the hierarchical clustering method and 200 hypoxia marker genes, hypoxic and non-hypoxic clusters were generated (Figure 3A). Patients in the hypoxia cluster exhibited higher immune cell infiltration by both the MCP-counter (Figure 3B) and TIMER2.0 (Figure 3C) methods (both *P* < 0.05). As illustrated in Figure 3D, the majority of immune checkpoint genes were expressed at higher levels in the hypoxia group (with the exception of indoleamine 2,3-dioxygenase 1, indoleamine 2,3-dioxygenase 2, and inducible T cell co-stimulator ligand).

### Generation of the Hypoxia-Related Score

**Figure 4:**
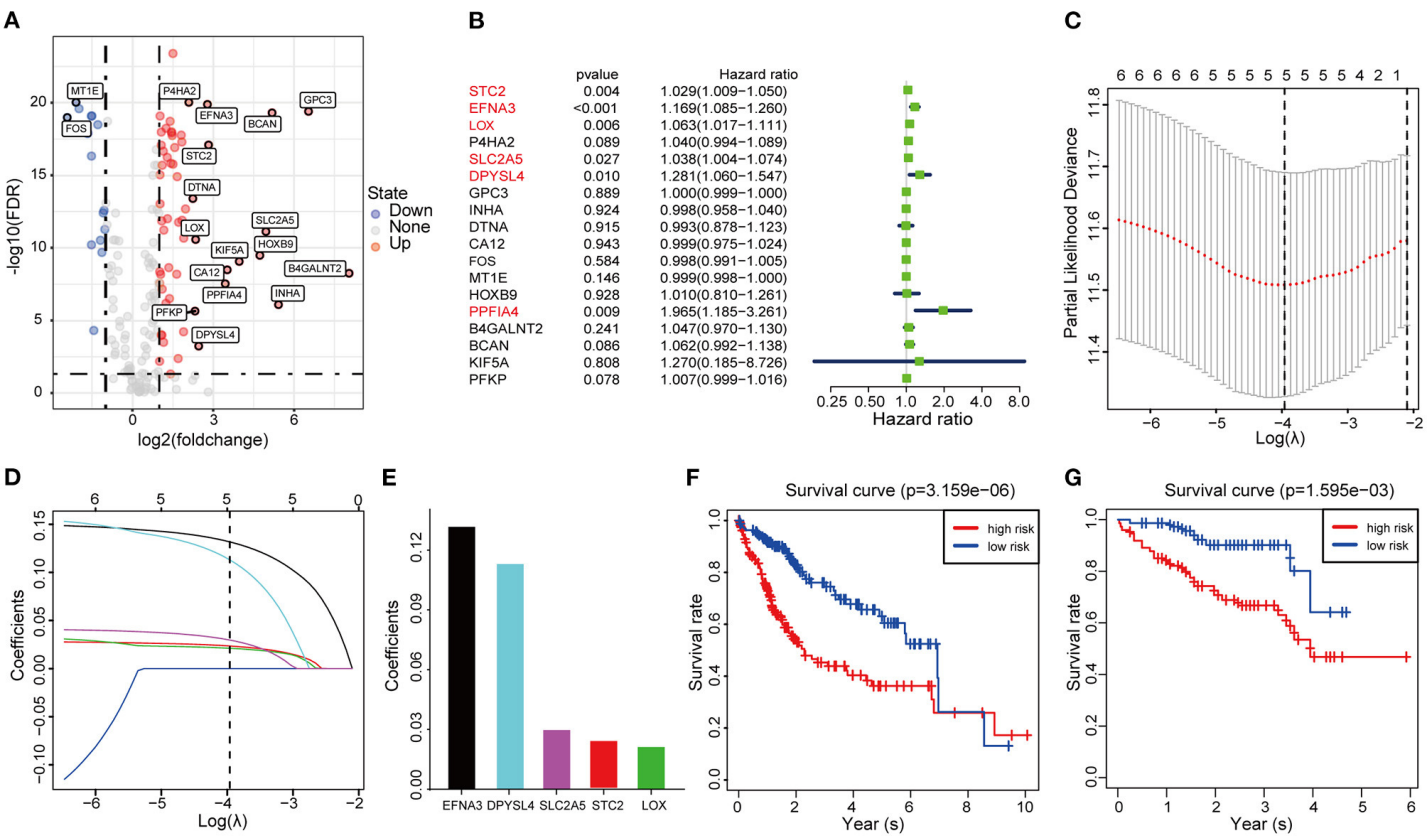
Construction and validation of the hypoxia-associated signature **(A)** Volcano plot of differentially expressed genes. **(B)** Univariable Cox regression analysis of differentially expressed genes. **(C)** Partial likelihood deviance for the lasso regression. **(D)** Lasso regression analysis. **(E)** Coefficients of the included genes. **(F,G)** Survival analysis based on TCGA cohort and ICGC cohort.

**Figure 5:**
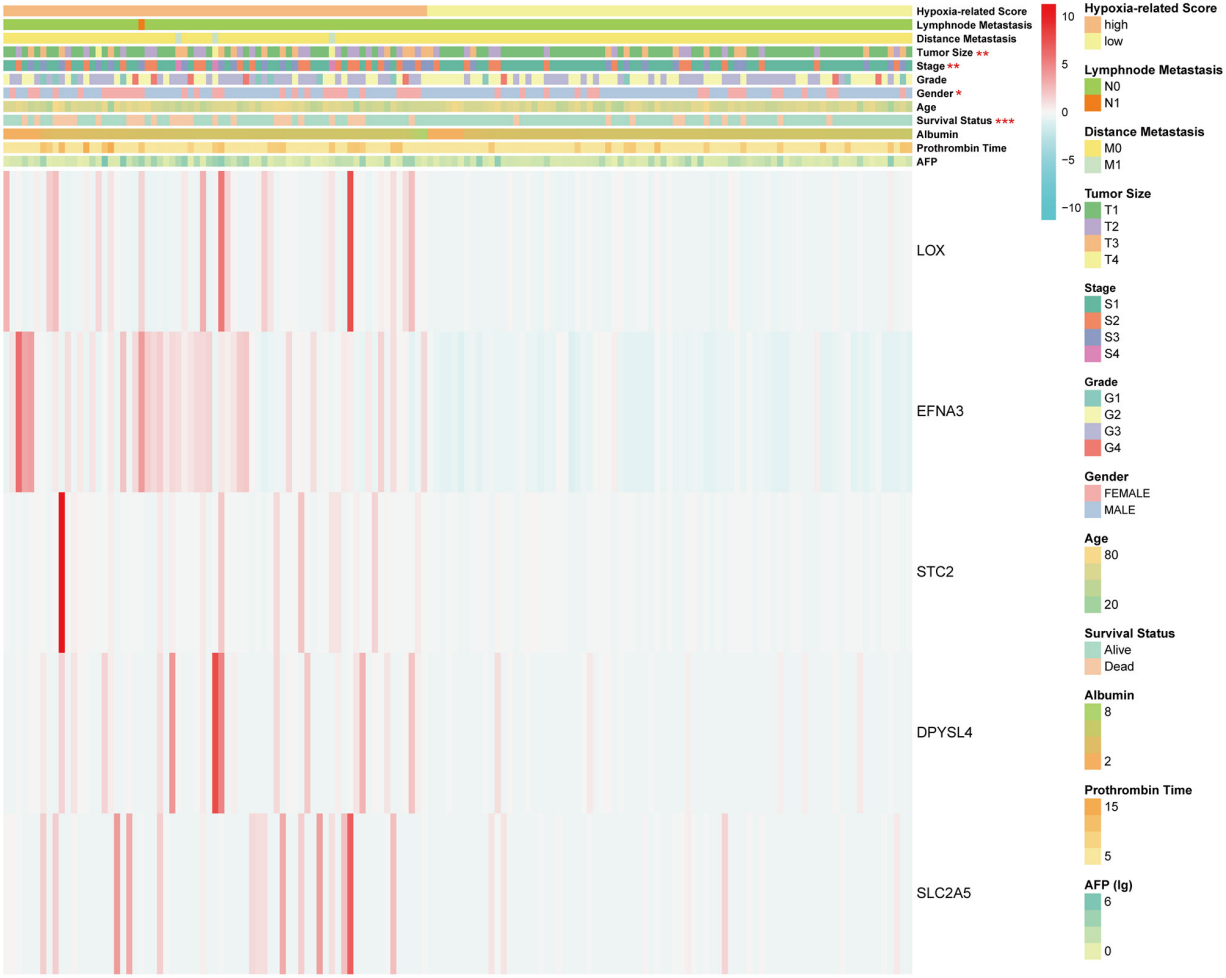
Heatmap of hypoxia-related score. *P < 0.05, **P < 0.01, ***P < 0.001. The units of some outcomes are following: AFP (ug/L), prothrombin time (seconds) and albumin (g/dL).

**Figure 6:**
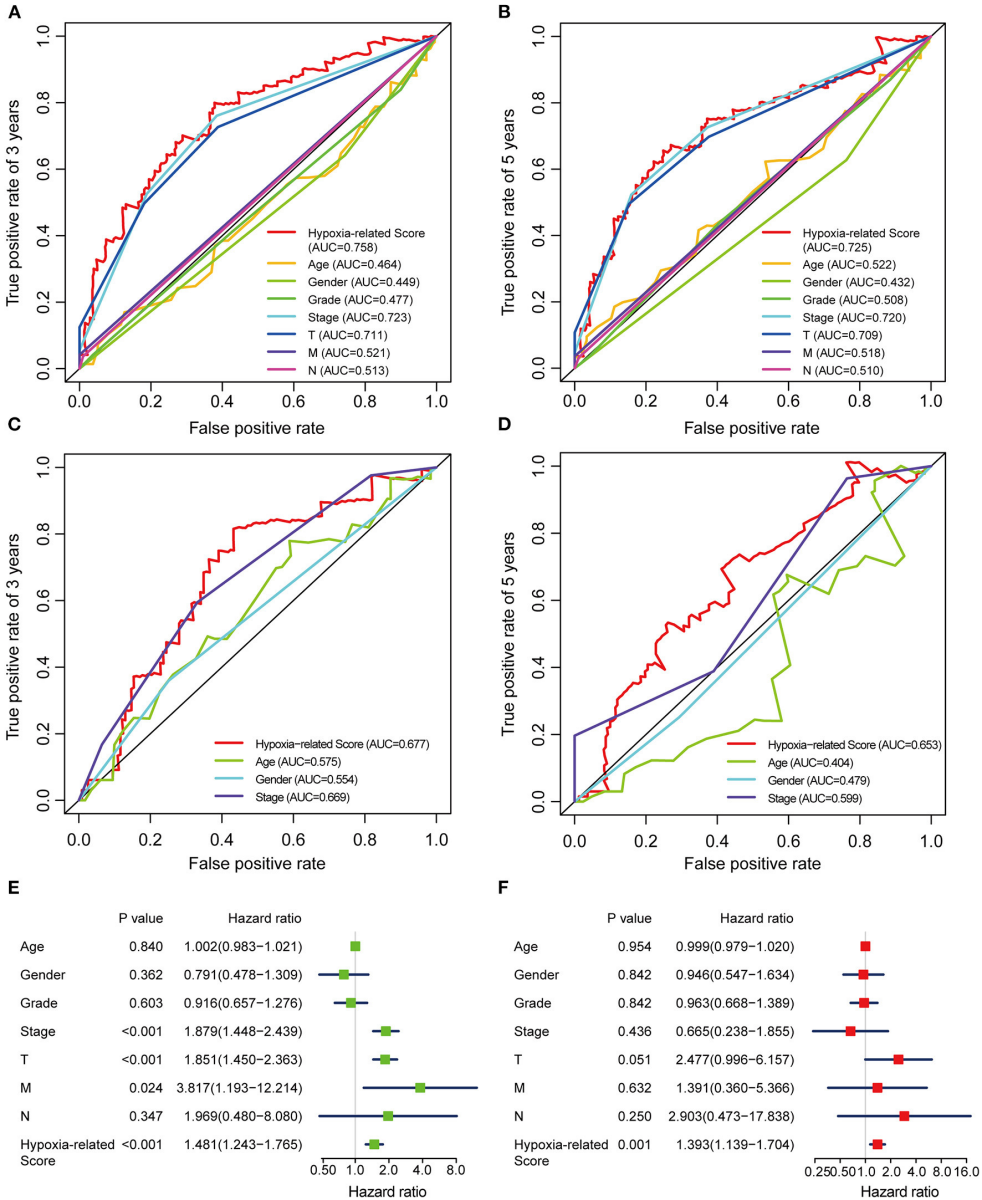
ROC and Cox regression analysis of the hypoxia-related score **(A,B)** ROC analysis of the hypoxia-related score based on TCGA cohort at 3 **(A)** and 5 **(B)** years. **(C,D)** ROC analysis of the hypoxia-related score based on ICGC cohort at 3 **(C)** and 5 **(D)** years. **(E)** Univariable Cox regression analysis. **(F)** Multivariable Cox regression analysis. “T” represents tumor size, “M” represents distance metastasis, “N” represents lymph node metastasis.

Considering the heterogeneity of hypoxia, we next quantified the hypoxic characteristics of different cases. To do this, we established a novel scoring system to evaluate the hypoxic characteristics of patients with immunosuppressive HCC. First, we performed differential expression analysis to identify differentially expressed hypoxia marker genes. Volcano plots indicated that 18 genes (metallothionein 1E; Fos proto-oncogene, AP-1 transcription factor subunit; prolyl 4-hydroxylase subunit alpha 2; ephrin A3; brevican; glypican 3; stanniocalcin 2; dystrobrevin alpha; lysyl oxidase; solute carrier family 2 member 5; kinesin family member 5A; homeobox B9; carbonic anhydrase 12; beta-1,4-N-acetyl-galactosaminyltransferase 2; PTPRF interacting protein alpha 4; inhibin subunit alpha; phosphofructokinase, platelet; and dihydropyrimidinase like 4) were eligible for further analysis (Figure 4A). Univariate Cox analysis (Figure 4B) and lasso regression analysis (Figures 4C,D) identified a score composed of five genes: ephrin A3, dihydropyrimidinase like 4, solute carrier family 2 member 5, stanniocalcin 2, and lysyl oxidase. The coefficients of these genes are presented in Figure 4E. Survival analysis of two cohorts demonstrated that the higher the score, the worse the overall survival (Figures 4F,G). Furthermore, the heatmap in Figure 5 indicates that the included genes were highly expressed in the hypoxia group. Hypoxia-related score were also significantly correlated with survival status, gender, tumor stage, and tumor size. In addition, when compared to other clinical parameters, the hypoxia-related score had the highest predictive value at both 3 and 5 years in two cohorts (Figures 6A–D). Univariate and multivariate Cox regression analysis indicated that the hypoxia-related score could serve as an independent prognostic marker in patients with immunosuppressive HCC (Figures 6E,F).

### Verification Using the GSE14520 Cohorts

**Figure 7:**
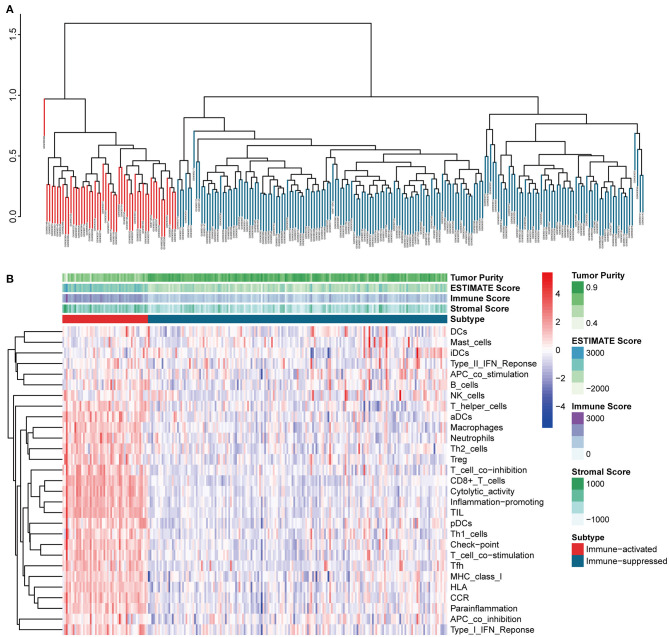
Identification of immune-associated clusters in the GEO cohort **(A)** Hierarchical clustering of immune-activated (red) and immune-suppressed (blue) clusters. Each branch in the tree diagram represents one case in the GSE14520 cohort. **(B)** Heatmap of immune-associated clusters, ssGSEA results, and ESTIMATE score.

**Figure 8:**
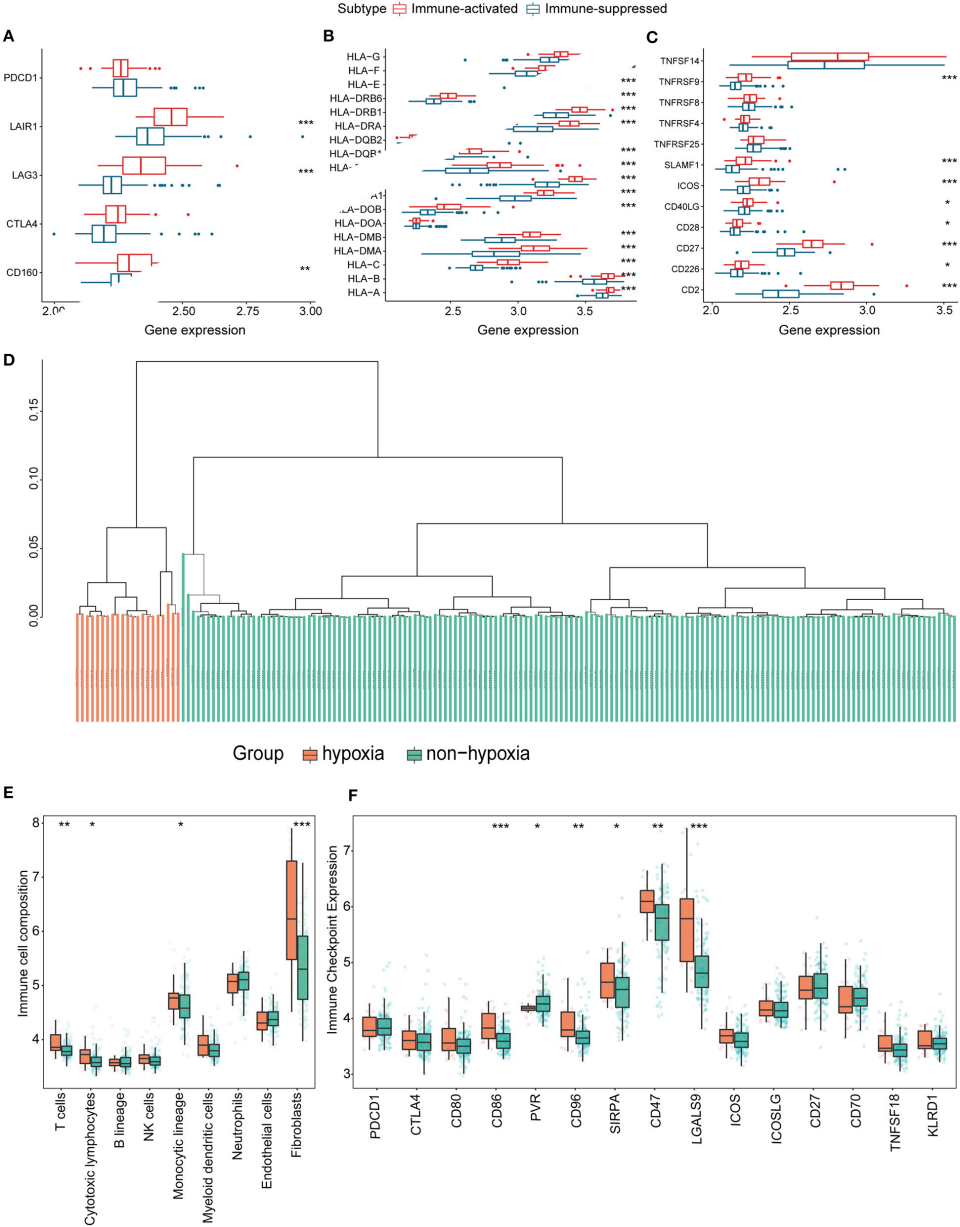
Identification and validation of hypoxia-associated clusters in the GEO cohort. *P < 0.05, **P < 0.01, ***P < 0.001. **(A–C)** Expression of T cell inhibitors **(A)**, major histocompatibility complexes **(B)**, and T cell stimulators **(C)** in each immune-associated cluster. **(D)** Hierarchical clustering tree of the hypoxia (orange) and non-hypoxia (green) clusters. **(E)** Immune cell infiltration in each hypoxia-associated cluster using the MCP-counter method. **(F)** Immune checkpoint gene expression in the hypoxia-associated clusters.

To validate the hypoxia-related subtype and verify the differences in the immune landscape, we used the independent GEO cohort (GSE14520) for patient clustering. The identified immunosuppressive cluster and hypoxia-related clusters were the same as those in the LIHC cohort (Figures 7, 8). Significant differences in immune cell infiltration were observed between the hypoxia and non-hypoxia groups, and CD86 molecule, poliovirus receptor (PVR), CD96 molecule, signal-regulatory protein alpha (SIRPA), CD47 molecule and galectin 9 (LGALS9) were significantly correlated with the hypoxia cluster.

## Discussion

The TME has significant influence on HCC (23), as it contains non-malignant cells that can promote tumor cells proliferation and metastasis. The immunosuppressive features of tumors not only induce cancer progression, but are also a challenge for effective immunotherapy (23). Consequently, the identification of TME-associated biomarkers for HCC is urgently needed. In this study, we identified patients with immunosuppressive HCC using 29 immune-related gene sets and hierarchical clustering. The proportions of patients in the immune-activated and immune-suppressed groups were consistent with the generally immunosuppressive nature of HCC. A significant difference in immune-associated gene expression was also observed, which verified the reliability of identifying patients with immunosuppressive HCC. Subsequently, we investigated the underlying relationship between hypoxia and immunosuppression. Hypoxia is an intrinsic characteristic of solid tumors because of an imbalance between the growth of tumor cells and their nutrient supply (24). The hypoxic TME stimulates HIF-driven transcription, which results in cell proliferation and metastasis (25). Meanwhile, it has been reported that hypoxia contributes to HCC cell proliferation, migration, and invasion (26), and accelerates malignant progression by impacting the crosstalk between stromal, tumor, and immune cells in the TME. For example, hypoxia can promote the recruitment of innate immune cells and interfere with the functions of adaptive immune cells (27). Therefore, we hypothesized that hypoxia could have an influence on patients with immunosuppressive HCC.

The hypoxia-associated genes employed in our research were all experimentally demonstrated to be upregulated in hypoxic conditions. Using these genes, we divided the patients with immunosuppressive HCC into two clusters. Hypoxic patients with immunosuppressive HCC exhibited higher immune cell infiltration and immune checkpoint expression, indicating an underlying correlation between hypoxia and the success of immunotherapy. A previous study (28) found that dynamic regulation of HIF1 activity is essential for normal B cell development. HIFs can also induce “don't-eat-me” signals that prevent phagocytosis and purinergic signaling, allowing tumor cells to evade immune surveillance (29). Based on the features of hypoxic patients with immunosuppressive HCC, hypoxia may be an effective biomarker to predict immunotherapeutic responses in patients with immunosuppressive HCC.

Immune subsets demonstrate different immunological functionality. It has been reported that B lymphocytes show numerous tumor-promoting characteristics (30). Another type of immune cells, NK cells, show protective and long-lasting immunity against diverse tumor types through direct cytotoxic activity or interacting with other immune cells (31). In our research, the infiltration of cells with immunosuppressive effect in hypoxia group like monocytic lineage and cancer-associated fibroblasts (both in TCGA and GEO) are higher than that in the non-hypoxia group, suggesting that hypoxia may aggravate the degree of immunosuppression. Classical monocytes mainly exhibit pro-tumor functions, such as differentiation into pro-tumor tumor-associated macrophages (TAMs), metastatic cell seeding, suppression of T cell function, recruitment of Tregs and so on (32). In terms of cancer-associated fibroblasts (CAFs), they are the main source of collagen-producing cells in the TME. CAFs provide mechanical support for tumor tissues and regulate the growth and invasion of tumor cells by remodeling the structure of the extracellular matrix (33). Therefore, we supposed that the more distinct infiltration of monocytes and CAFs played a crucial role in the immunosuppressive TME caused by hypoxia. Even the infiltration of cells with anti-tumor immune response like T cells (both in TCGA and GEO) are also higher in the hypoxia group, the function of T cell may be weakened due to hypoxia. It has been reported that hypoxia, adenosine, lactic acid and low pH impaired the ability of dendritic cells to stimulate T cell responses (34). The different infiltration level of the cells and differentially expressing immune checkpoint genes confirmed the difference of TME and immunotherapeutic response between two groups.

Furthermore, we constructed a novel scoring system (the hypoxia-associated score) to evaluate the hypoxic characteristics of patients with immunosuppressive HCC. The score included five genes (ephrin A3, dihydropyrimidinase like 4, solute carrier family 2 member 5, stanniocalcin 2, and lysyl oxidase) that were all highly expressed in the high-risk group and significantly correlated with worse prognosis. So far, only two of these genes *(STC2* and *LOX*) have been experimentally verified. Umezaki et al. concluded that LOX induced epithelial-mesenchymal transition and could be used to predict intrahepatic metastasis in HCC (35). Wang et al. (36) reported that high expression of STC2 may be associated with HCC occurrence, development, and prognosis. Although no evidence has been found to support the three other genes, they may be novel predictors in HCC. In addition, through the ROC plot and Cox regression analysis, the hypoxia-associated score presented their clinical potential and may serve as an independent predictive biomarker of HCC.

To our knowledge, this is the first study to identify a hypoxia-associated subtype of patients with immunosuppressive HCC. In contrast to a previous study (24), our study presented the following different points. Firstly, the sources of hypoxia-associated genes were distinct. Their study identified the relevant genes by differential expression analysis while we employed the hypoxia-associated genes from Molecular Signature Database. The hypoxia-associated gene set in our research was identified from four datasets (GSE18494, GSE30797, GSE33607, GSE9649) and validated from one dataset (GSE14762), which made the hypoxia-associated genes involved in our research more reliable and specific. Secondly, about 90% patients in our study were identified as the immunosuppressive cluster, which presented the different features (higher tumor purity, lower immune score, higher gene expression of immune checkpoints, and more antigen presentation) compared with the immune-activated cluster. We believe that it is necessary to identify the specific immunosuppressive cluster as the topic for a future study in HCC. Furthermore, the reliable hypoxia-associated genes and comprehensive methodology used in our study enabled the identification of a robust signature. We propose that this signature represents a novel biomarker to predict the immunotherapeutic responses of these patients in the clinic. Nevertheless, there are some limitations to our study. First, many other complicated mechanisms influence the development and progression of HCC, and there may be an intrinsic weakness in using a single characteristic to construct a predictive model. Second, no more available clinical information can be found in TCGA database, so the established prognostic model cannot take into account the clinical environment. Further, our evidence is based on bioinformatics methodology and should be considered preliminarily until its verification through more wet lab experiments and clinical trials.

In conclusion, our study illustrates the crucial role of hypoxia in patients with immunosuppressive HCC. The defined hypoxia-associated subtype may help reveal regulatory mechanisms between hypoxia and the immunosuppressive microenvironment, and its related score exhibits potential implications for future predictive models.

## Data Availability Statement

Publicly available datasets were analyzed in this study. This data can be found here: TCGA database (https://portal.gdc.cancer.gov/), GEO database (https://www.ncbi.nlm.nih.gov/geo/), and ICGC database (https://dcc.icgc.org/).

## Author Contributions

ZM and SZ designed the manuscript. ZM and DL wrote and completed the manuscript. ZM and DR completed the data download and analysis. All authors contributed to the article and approved the submitted version.

## Conflict of Interest

The authors declare that the research was conducted in the absence of any commercial or financial relationships that could be construed as a potential conflict of interest.
